# Supplementary material for: Sex Classification by Resting State Brain Connectivity
Source: Cereb Cortex. 2019 Jun 28;30(2):824–35. doi: 10.1093/cercor/bhz129 (PMC7444737; doi:10.1093/cercor/bhz129)
Supplement: Weis_CerCor-2018-01213_supp_fig_legend_bhz129 [file weis_cercor-2018-01213_supp_fig_legend_bhz129.docx]

**Supplementary Figure 1:** ROI based classification accuracies for within sample cross validation in sample 4. While the classification accuracies are lower than for sample 1, the spatial distribution is comparable to the within-sample cross validation in sample 1.
