# Supplementary figures and images for: Sex Classification by Resting State Brain Connectivity
Source: Cereb Cortex. 2019 Jun 28;30(2):824–35. doi: 10.1093/cercor/bhz129 (PMC7444737; doi:10.1093/cercor/bhz129)

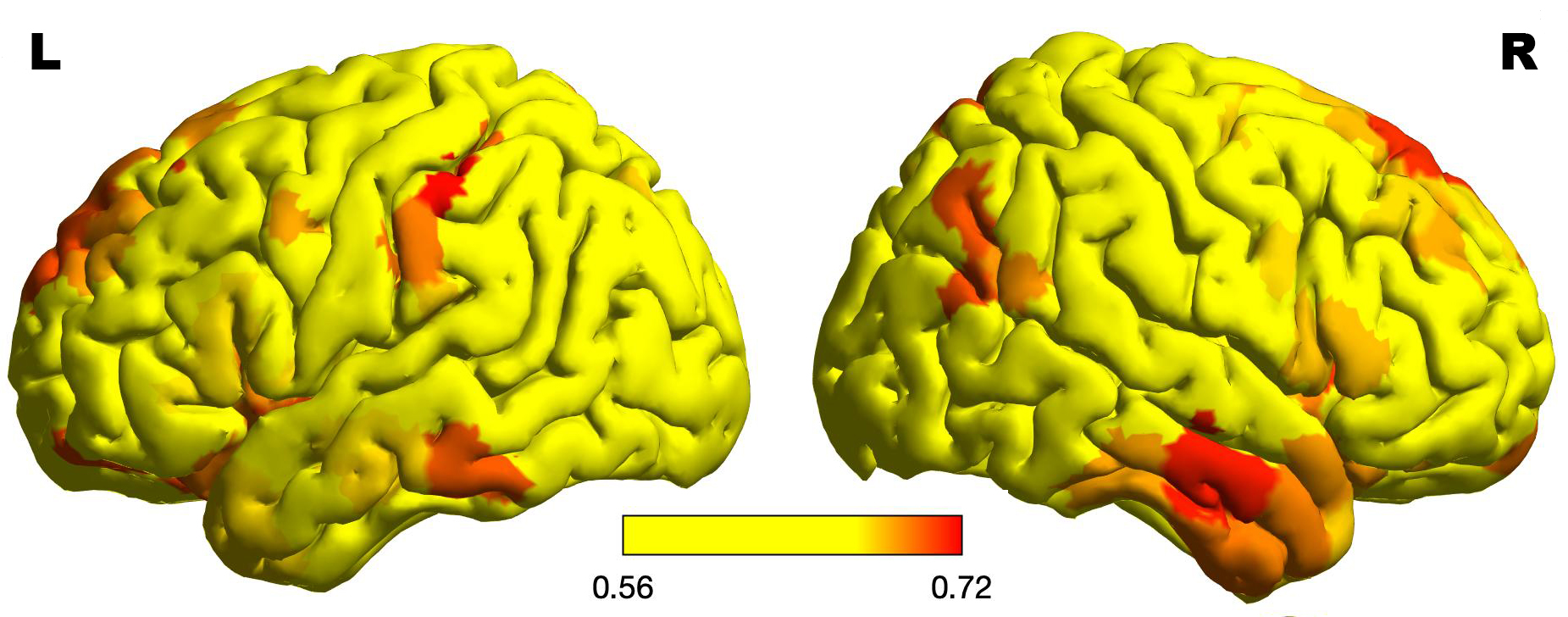

Supplement: Weis_etal_Supplementary_R1_bhz129 [file weis_etal_supplementary_r1_bhz129.jpeg]
